# Ubiquitination and Degradation of CFTR by the E3 Ubiquitin Ligase MARCH2 through Its Association with Adaptor Proteins CAL and STX6

**DOI:** 10.1371/journal.pone.0068001

**Published:** 2013-06-20

**Authors:** Jie Cheng, William Guggino

**Affiliations:** Department of Physiology, Johns Hopkins University, School of Medicine, Baltimore, Maryland, United States of America

## Abstract

Golgi-localized cystic fibrosis transmembrane conductance regulator (CFTR)-associated ligand (CAL) and syntaxin 6 (STX6) regulate the abundance of mature, post-ER CFTR by forming a CAL/STX6/CFTR complex (CAL complex) that promotes CFTR degradation in lysosomes. However, the molecular mechanism underlying this degradation is unknown. Here we investigated the interaction of a Golgi-localized, membrane-associated RING-CH E3 ubiquitin ligase, MARCH2, with the CAL complex and the consequent binding, ubiquitination, and degradation of mature CFTR. We found that MARCH2 not only co-immunoprecipitated and co-localized with CAL and STX6, but its binding to CAL was also enhanced by STX6, suggesting a synergistic interaction. In vivo ubiquitination assays demonstrated the ubiquitination of CFTR by MARCH2, and overexpression of MARCH2, like that of CAL and STX6, led to a dose-dependent degradation of mature CFTR that was blocked by bafilomycin A1 treatment. A catalytically dead MARCH2 RING mutant was unable to promote CFTR degradation. In addition, MARCH2 had no effect on a CFTR mutant lacking the PDZ motif, suggesting that binding to the PDZ domain of CAL is required for MARCH2-mediated degradation of CFTR. Indeed, silencing of endogenous CAL ablated the effect of MARCH2 on CFTR. Consistent with its Golgi localization, MARCH2 had no effect on ER-localized ΔF508-CFTR. Finally, siRNA-mediated silencing of endogenous MARCH2 in the CF epithelial cell line CFBE-CFTR increased the abundance of mature CFTR. Taken together, these data suggest that the recruitment of the E3 ubiquitin ligase MARCH2 to the CAL complex and subsequent ubiquitination of CFTR are responsible for the CAL-mediated lysosomal degradation of mature CFTR.

## Introduction

Cystic fibrosis (CF) is a lethal recessive genetic disorder caused by loss-of-function mutations in the CF transmembrane conductance regulator (CFTR) gene [1–3]. The biosynthesis, endocytosis, recycling, and quality control of CFTR are tightly regulated by the ubiquitination system [4–11]. CFTR molecules that are misfolded early in their biosynthesis in the ER or during later trafficking to the cell periphery are ubiquitinated and degraded by quality control systems in the proteasomes and lysosomes, respectively [12–15]. Within the ubiquitination system, E3 ubiquitin ligases demonstrate specificity in selecting ubiquitin substrates; these ligases often require accessory proteins to facilitate their binding to target proteins. In the case of CFTR, multiple E3 ubiquitin ligase complexes (CHIP, RMA1, gp78, HRD1 and associated accessory proteins) are involved in its ER-associated degradation (ERAD) [7,16,17]. CHIP is also involved in the peripheral quality control of CFTR but with a distinct set of chaperones, co-chaperones and E2 ubiquitin conjugating enzyme [12]. E3 ligase c-Cbl and the adaptor protein Dab2 complexes have been implicated in the endocytosis and recycling of CFTR in early endosomes [4,5]. Thus far, several E3 ligases have been shown to participate in CFTR trafficking from the ER to the plasma membrane. However, no Golgi-localized E3 ubiquitin ligases have yet been shown to ubiquitinate CFTR.

Golgi-localized CFTR-associated ligand (CAL) and syntaxin 6 (STX6) regulate the abundance of mature, post-ER CFTR by forming CAL/STX6/CFTR complexes (CAL complexes) that promote the degradation of CFTR in lysosomes [18,19]. RNA silencing of CAL or STX6 increases the production of mature CFTR, whereas overexpression of CAL or STX6 reduces CFTR abundance [19–21]. Synthetic CAL inhibitors that selectively disrupt the interaction of CAL with CFTR increase the surface expression of both wild-type and ΔF508 CFTR [22,23]. How the binding to Golgi-localized CAL complexes promotes the lysosomal targeting of CFTR is, however, still unknown. Since ubiquitination systems are widely utilized in tagging proteins, including CFTR, for degradation, it is of interest that two Golgi-localized E3 ubiquitin ligase membrane-associated RING-CH (MARCH) family proteins, MARCH2 and MARCH3, were recently shown to interact with STX6 [24,25]. Homologs of the MARCH E3 ubiquitin ligase family were first identified as K3 and K5 viral proteins related to immune-evasion strategies used by Kaposi’s sarcoma-associated herpesvirus (KSHV) [26–28]. K3 and K5 viral ubiquitin E3 ligases degrade a number of host cell-surface receptors, including MHC class I molecules, in lysosomes [26]. Cellular MARCH family proteins ubiquitinate and regulate several membrane receptors and their trafficking [29,30]. For examples, MARCH1 ubiquinates HLA-DR and promotes its lysosomal degradation [31,32]. MARCH1 also down-regulates TFRC, CD86, and FAS [29,30,33]. MARCH2 has been reported to reduce the surface expression of CD86 and the transferrin receptor TFRC [30] and regulate cell surface β(2)-adrenergic receptor expression [34].

In this paper, we explore the possible role of the MARCH2 ubiquitin E3 ligase in the lysosomal degradation of mature CFTR and its interaction with the CAL complex. We first assessed the potential interaction and co-localization of MARCH2 with the CAL complex. We then tested the ability of MARCH2 to foster the ubiquination and degradation of CFTR and its dependence on CAL complex. Furthermore, we used siRNA-mediated silencing to investigate the role of endogenous MARCH2 in a CF bronchial epithelial cell line. Our results provide a clearer picture of the molecular mechanism underlying the CAL complex-mediated lysosomal degradation of CFTR by linking an E3 ligase, MARCH2, to the CAL complex.

## Materials and Methods

### Cell Culture and Chemicals

Human embryonic kidney cells (HEK293), obtained from the American Type Tissue Culture (ATCC), were maintained in DMEM: F12 medium (1:1) with 20 mM L-glutamine, 100 unit/ml penicillin, 100 µg/ml streptomycin, and 10% fetal calf serum. CFBE41o-cells stably transfected with wildtype CFTR (CFBE-CFTR) (a gift of Dr. Dieter Gruenert, California Pacific Medical Center Research Institute, San Francisco) were maintained in MEM with 20 mM L-glutamine, 100 unit/ml penicillin, 100 µg/ml streptomycin, 300 µg/ml hygromycin B, and 10% fetal calf serum. Media and other components were purchased from Invitrogen (Grand Island, NY). HEK293 cells were transfected using Lipofectamine 2000 (Invitrogen, Grand Island, NY) according to the manufacturer’s instructions. Unless indicated, all chemicals were purchased from Sigma-Aldrich (St. Louis, MO).

### Plasmids and Plasmid Construction

HA-tagged (HA-MARCH2) and myc-tagged MARCH2 (myc-MARCH2) were constructed by PCR amplification of cDNA obtained from Open Biosystems (Huntsville, AL) and subcloning into *Sal*I/*Not*I sites of the HA-tagged or myc-tagged pRK5 mammalian expression vector (pRK5-HA or pRK5-myc) (gifts of Drs. A. A. Lanahan and P. F. Worley, the Johns Hopkins University). The RING mutant of MARCH2 (HA-MARCH2-RINGmut) where Zn2+-binding cysteine residues of HA-MARCH2 RING domain were substituted with serine residues (C64S/C67S) was constructed by using a QuikChange^TM^ site-directed mutagenesis kit (Stratagene, La Jolla, CA). The PDZ motif deletion mutant of MARCH2 (HA-ΔTPV-MARCH2 and myc-ΔTPV-MARCH2) where C-terminal three residues (TPV) were deleted were constructed by using a QuikChange^TM^ site-directed mutagenesis kit (Stratagene, La Jolla, CA). His_6_-ubiquitin construct was a gift from Dr. Dirk Bohmann (University of Rochester). pCMV-CFTR was constructed by inserting the coding region of human CFTR in the mammalian expression vector pcDNA3.1 vector (a gift from Dr. Garry Cutting, the Johns Hopkins University). All of the constructs above were sequence-verified in both directions by automated fluorescent sequencing (by the Johns Hopkins University Biosynthesis and Sequencing Facility). All other plasmid constructs used were previously published [18].

### siRNA Silencing

Annealed, double-stranded siRNA was transfected into HEK293 and CFBE-CFTR cells using INTERFERin transfection reagent (Polyplus, New York, NY) according to the manufacturer’s instructions. MARCH2 siRNA1 (Hs_MARCH-II_1 FlexiTube siRNA), MARCH2 siRNA2 (Hs_MARCH-II_3 FlexiTube siRNA), CAL siRNA (Hs_GOPC_3_HP siRNA) and negative control siRNA (AllStars negative control siRNA) were purchased from Qiagen (Valencia, CA). At 24 h after siRNA transfection, DNA plasmids were transfected using Lipofectamine 2000 (Life Technologies, Grand Island, NY) as indicated.

### Immunoprecipitation and Immunoblot

Cells were harvested and processed as described previously (Cheng et al., 2002). In brief, they were solubilized in lysis buffer (150 mM NaCl, 50 mM Tris-HCl, pH 7.4, 1% Nonidet P-40, and complete protease inhibitor (Roche Applied Science, Indianapolis, IN). The cell lysates were centrifuged at 14,000 rpm for 15 min at 4 °C in a micro-centrifuge (Eppendorf, Hauppauge, NY) to pellet insoluble material. The protein concentrations of the supernatants were quantified with a BCA protein assay kit (Thermo Scientific, Rockford, IL). For immunoprecipitation experiments, 500 µg supernatants was incubated with anti-HA affinity matrix (Roche Applied Science, Indianapolis, IN) at 4^o^C overnight. The normalized supernatants were subjected to SDS-PAGE on 5% or 4-15% Ready-gel (BioRad, Hercules, CA) and immunoblot analysis, followed by ECL (Amersham Biosciences, Pittsburgh, PA). The chemiluminescence signal on the polyvinylidene difluoride membrane was directly captured by a FujiFilm LAS-3000 plus system with 3,200,000-pixel cooled super CCD camera having a linearity of 4 orders of magnitude. Quantification was carried out within the linear range using the Image Gauge version 3.2 software (FujiFilm, Stamford, CT). CFTR protein level was normalized by the densitometry of GAPDH presented in the same lane on the SAS-PAGE and normalized again by the corresponding control samples to obtain the value of percent of control (%). CFTR was detected with mouse monoclonal antibody 217 (1:2000; UNC, Chapel Hill, NC). GAPDH was detected with a mouse monoclonal antibody (1:5000; United States Biological, Marblehead, MA). Mono- and poly-ubiquitinated proteins were detected with a mouse monoclonal anti-ubiquitinated proteins antibody, clone FK2 (Millipore, Billerica, MA). GFP-tagged proteins were detected with a rabbit polyclonal anti-GFP antibody (1:1000; BD Biosciences, Boston, MA). HA-tagged proteins were detected with a mouse monoclonal anti-HA antibody (1:2000; Roche Applied Science) or a rabbit polyclonal anti-HA antibody (1:500; Roche Applied Science). The expression of myc-tagged proteins was detected with a mouse monoclonal anti-myc antibody (1:2000; Roche Applied Science).

### In Vivo Ubiquitination Assays

In vivo ubiquitination assays were performed as described [35], except that the HEK293 cells were first transfected with CFTR (pCMV-CFTR) and His6-ubiquitin. After 24 h, the cells were transfected again with HA-MARCH2 in the presence of 400 nM Bafilomycin 1A.

### Confocal Microscopy

Cells were placed on glass coverslips 1 day before transfection. One day after transfection, the cells were fixed in 4% paraformaldehyde and permeabilized in 0.2% Nonidet P-40. Nonspecific binding sites were blocked with 5% normal goat serum. The cells were stained with primary antibodies in 5% normal goat serum. HA-tagged proteins were detected with a mouse monoclonal anti-HA antibody (1:2000; Roche Applied Science). myc-tagged proteins were detected with a rabbit polyclonal anti-myc antibody (1:1000; Santa Cruz biotechnology, Santa Cruz, California). GFP-tagged proteins were detected with a rabbit polyclonal anti-GFP antibody (1:2000; Roche Applied Science). Golgi 160 was detected with a rabbit polyclonal anti Golgin 160 (gift of Dr. C Machamer, the Johns Hopkins University). Cells were then washed with 1% bovine serum albumin and incubated with goat Cy3-conjugated secondary antibody (1:200; Jackson ImmunoResearch, West Grove, PA) or goat anti-rabbit Alexa Fluor 647-conjugated secondary antibody (1:200; Invitrogen, Grand Island, NY) or goat anti-mouse Alexa Fluor 594-conjugated secondary antibody (1:200; Invitrogen, Grand Island, NY) in 1% normal goat serum. The specimens were washed, mounted, and viewed on an LSM510-Meta laser confocal microscope (Zeiss).

### Short-Circuit Current Measurements

Short-circuit current (Isc) measurements were performed in six-channel Easy-Mount chambers system (Physiologic Instruments, San Diego, CA) that accepts Snapwell filters (Corning Costar, Acton, MA; 3407). Isc was measured with a VCCMC6 multichannel voltage-current clamp amplifier (Physiologic Instruments) in the voltage-clamp mode. Data were acquired on a 1.71-GHz PC running Windows XP (Microsoft, Redmond, WA) equipped with DI-720 (DATAQ Instruments, Akron, OH) with software Acquire and Analyze version 2.3.159 (Physiologic Instruments). Cells were cultured to confluence on Snapwell filters before measurement. The cell monolayers were bathed on both sides with a solution containing (in mM): 115 NaCl, 25 Na-gluconate, 5K-gluconate, 1.2 MgCl_2_, 1.2 CaCl_2_, 10 d-glucose, and 10 HEPES (pH 7.4 with NaOH). The mucosal side was replaced with a low Cl_ solution containing (in mM): 139 Na-gluconate, 1.2 NaCl, 5 K-gluconate, 1.2 MgCl_2_, 1.2 CaCl_2_, 10 d-glucose, and 10 HEPES (pH 7.4 with NaOH). The solution was constantly circulated, maintained at 37°C, and bubbled gently with air. Amiloride (10 µM) was added to the mucosal solution, and after stabilization, forskolin (10 µM) was added to the serosal chamber followed by the CFTR channel inhibitor CFTRinh-172 (1 µM).

### Statistical Analysis

The data are presented as the means +/- standard errors. Statistical significance was determined by Student’s *t*-test. We assigned significance at p < 0.05.

## Results

### Localization and interaction of MARCH2 with CAL and STX6

**Figure 1:**
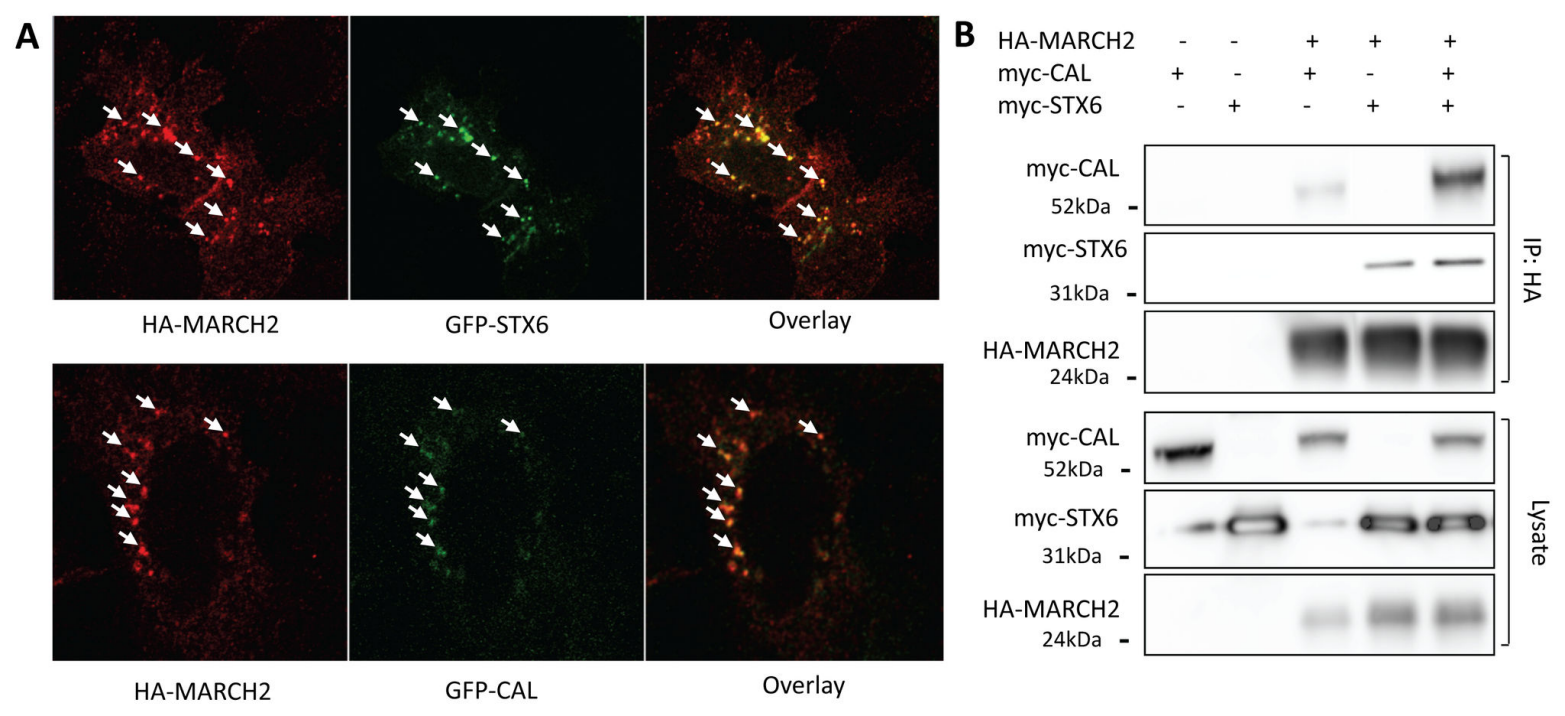
Co-localization and interaction of MARCH2 with STX6 and CAL. (**A**) HEK293 cells grown on coverslips were co-transfected with 3µg GFP-STX6 or 3µg GFP-CAL and 1µg HA-MARCH2. Twenty-four hours after transfection, cells were fixed and subjected to indirect fluorescent immunocytochemical staining with an anti-HA monoclonal antibody followed by Cy3-conjugated secondary antibody. HA-MARCH2 appears in red and GFP-STX6 and GFP-CAL are green. Arrow heads point to colocalization. (**B**) HEK293 cells were co-transfected with 3µg myc-CAL, 3µg myc-STX6, and 1µg HA-MARCH2 as indicated. After 48 h, cell lysates were harvested and immunoprecipitated with an anti-HA affinity matrix. Cell lysates and immunoprecipitated materials were subjected to immunoblot analysis with Myc or HA antibodies. Data shown are representative of at least three independent experiments.

Since MARCH2 and CAL are both localized to the Golgi and Golgi-derived vesicles (Figure S1) [18,24], we examined the possible co-localization of these two proteins. Given the lack of high-quality antibodies against MARCH2, we instead co-transfected HEK293 cells with epitope-tagged HA-MARCH2 and GFP-CAL in order to examine their co-localization. At 24 h post-transfection, the cells were fixed, permeablized, and subjected to indirect fluorescent immunostaining. Laser confocal microscopy showed that HA-MARCH2 indeed co-localized with GFP-CAL (Figure 1A). HA-MARCH2 also co-localized with GFP-STX6, confirming an earlier observation [24] (Figure 1A).

We then examined the interactions of MARCH2 with CAL and STX6 by immunoprecipitation after transfecting HEK293 cells with HA-MARCH2, myc-CAL, and myc-STX6 constructs. HA-MARCH2 was co-immunoprecipitated with myc-CAL and myc-STX6 when it was co-expressed with myc-CAL and myc-STX6, respectively (Figure 1B). Interestingly, when the cells were triple-transfected with HA-MARCH2, myc-CAL, and myc-STX6 constructs, the presence of myc-STX6 increased the amount of myc-CAL immunoprecipitated by HA-MARCH2, suggesting that a synergistic interaction and complex formation occurred between MARCH2, CAL, and STX6 (Figure 1B).

**Figure 2:**
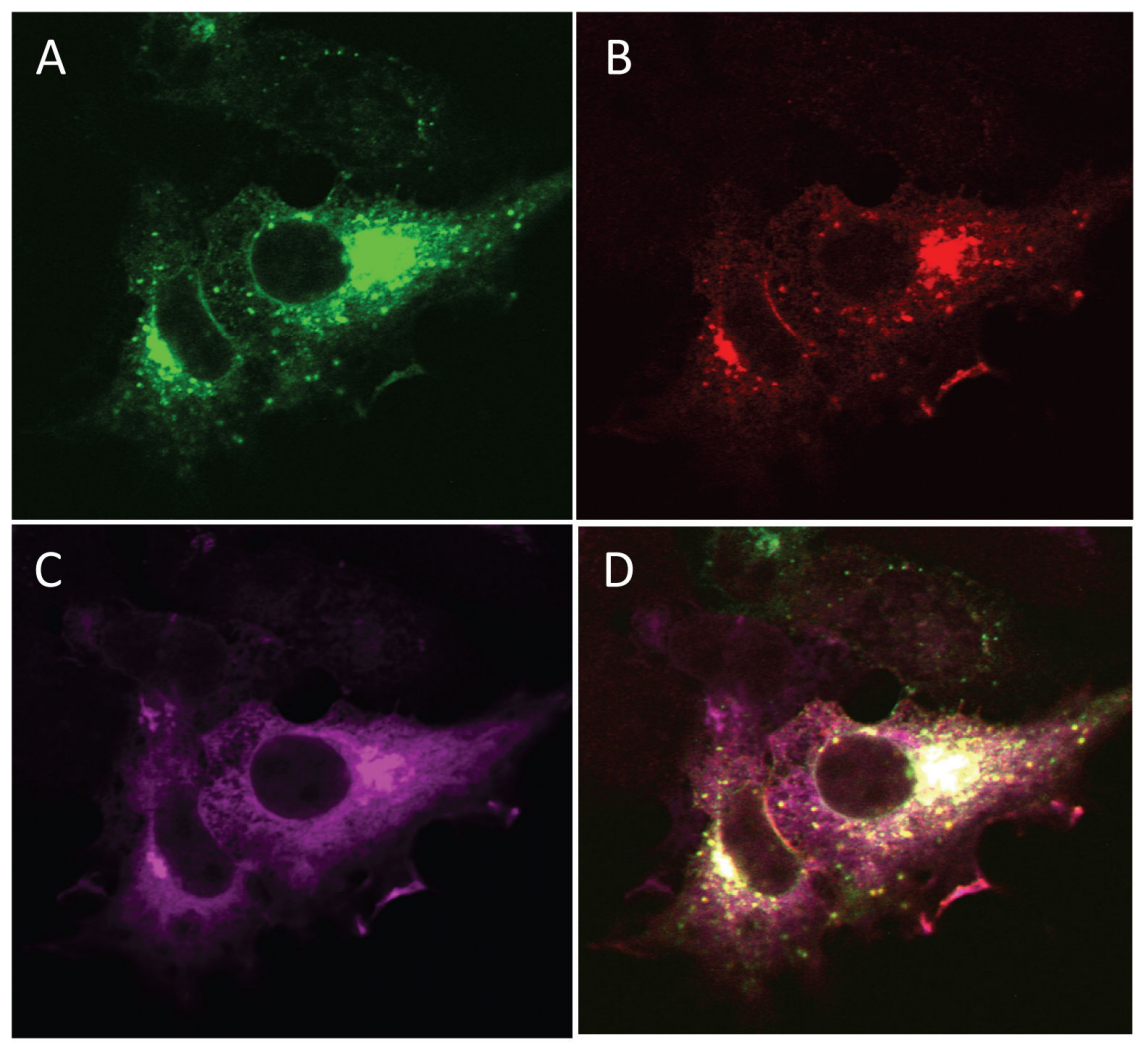
Co-localization of MARCH2, STX6, and CAL. HEK293 cells grown on coverslips were co-transfected with 3µg GFP-STX6, 3µg myc-CAL, and 1µg HA-MARCH2. After 24 h, cells were fixed and subjected to indirect fluorescent immunocytochemical staining with an anti-myc rabbit polylonal antibody and an anti-HA mouse monoclonal antibody followed by goat anti-rabbit Alexa Fluor 647-conjugated secondary antibody and goat anti-mouse Alexa Fluor 594-conjugated secondary antibody. (**A**) GFP-STX6 appears in green. (**B**) HA-MARCH2 appears in red. (**C**) myc-CAL appears in magenta. (**D**) Overlay of GFP-STX6, HA-MARCH2 and myc-CAL. Data shown are representative of at least three independent experiments.

To determine whether all three proteins (MARCH2, CAL, and STX6) are co-localized to the same subcellular compartment, we performed triple-transfection of MARCH2, CAL and STX6 constructs in HEK293 cells, then fixed and permeabilized the cells 24 h later and subjected them to indirect fluorescent immunostaining. Laser confocal microscopy demonstrated the co-localization of all three proteins when co-expressed (Figure 2). These data indicate the association of ubiquitin E3 ligases MARCH2 with the CAL complex and suggest a possible role of MARCH2 in the CAL-mediated lysosomal degradation of CFTR.

### Degradation of CFTR by MARCH2

**Figure 3:**
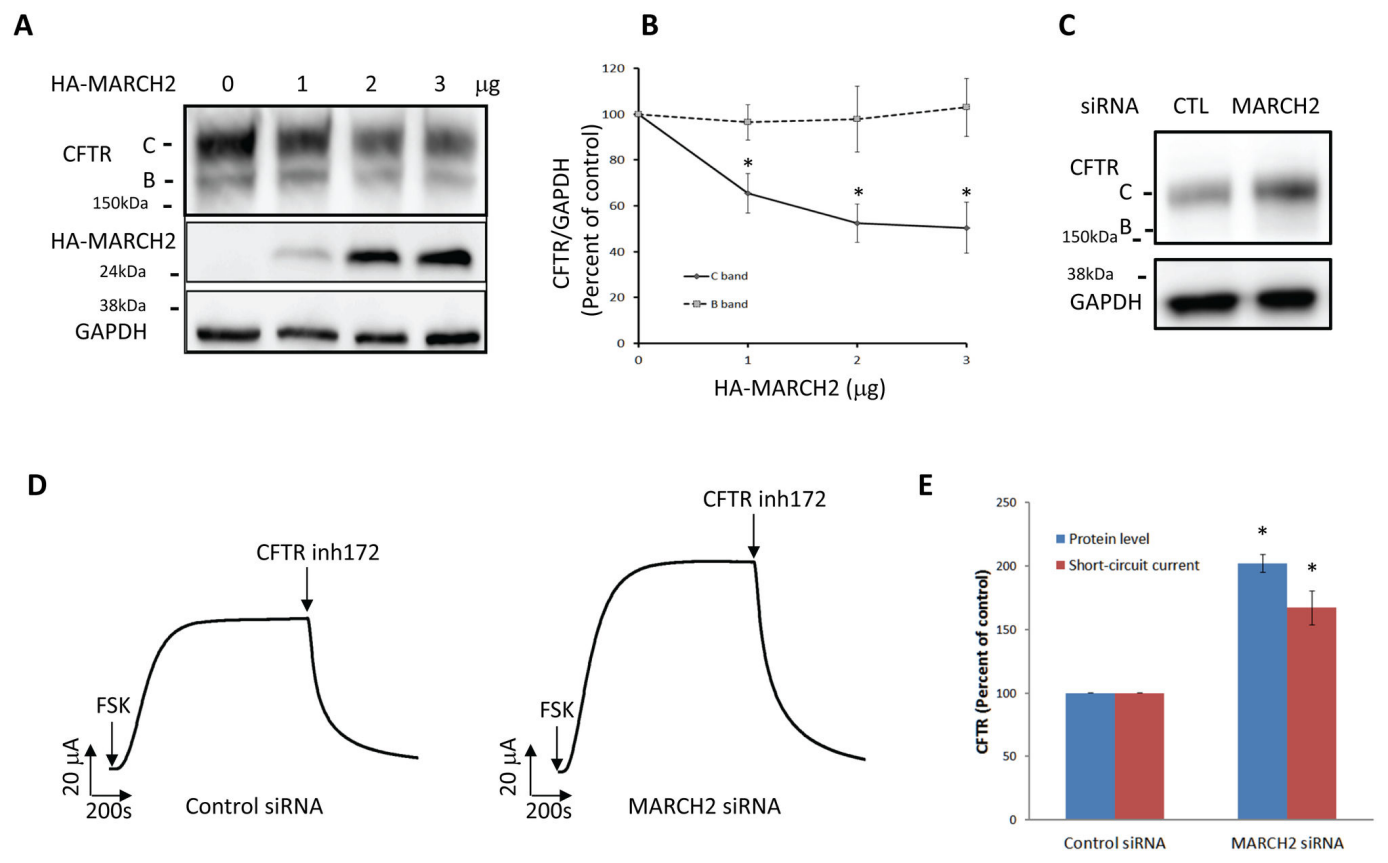
Effect of overexpression and silencing of MARCH2 on CFTR. (**A**) HEK293 cells were co-transfected with 3µg GFP-CFTR and increasing amounts of HA-MARCH2 (1µg, 2µg, and 3µg as indicated). After 48 h, cell lysates were harvested and subjected to immunoblot analysis with anti-CFTR, anti-HA, and anti-GAPDH antibodies. (**B**) Densitometric analysis of CFTR in (A). CFTR is normalized to GAPDH. Values are presented as mean +/- S.E. *, p<0.05 versus control (n=3) (**C**) CFBE-CFTR cells were transfected with 20 nM of negative control (CTL) siRNA (AllStars negative control siRNA) or MARCH2 siRNA (Hs_MARCH-II_1 FlexiTube siRNA) as indicated. After 72 h, cell lysates were collected and subjected to immunoblot analysis with the indicated antibodies. (**D**) CFBE-CFTR cells grown on permeable supports were transfected with 20 nM of negative control siRNA (AllStars negative control siRNA) or MARCH2 siRNA (Hs_MARCH-II_1 FlexiTube siRNA) as indicated. Seventy-two hours after transfection, cells were mounted on the Ussing chamber setup, where short-circuit currents were measured as described in *Materials and Methods*. In the graph, 10 µM forskolin (FSK) and 1 µM CFTRinh-172 were added as indicated. (**E**) Densitometric analysis of CFTR protein level in (C) and summary of the short-circuit currents in (D). Values are presented as mean +/- S.E. *, p<0.05 versus control (n=3).

Since the overexpression of CAL and STX6 leads to the degradation of CFTR in lysosomes [19,21], we tested the effect of overexpressing MARCH2 on CFTR degradation. CFTR and HA-MARCH2 were expressed in HEK293 cells, and at 48 h post-transfection, total protein lysates were harvested and the CFTR protein level measured by immunoblot analysis. We found that overexpression of HA-MARCH2 indeed reduced the CFTR protein, in a dose-dependent manner (Figure 3A and B). To confirm the role of endogenous MARCH2 in human bronchial epithelial cells, we examined the effect of siRNA-mediated silencing of MARCH2 gene on the expression of CFTR. Previously, silencing of CAL and STX6 in a CF bronchial epithelial cell line (CFBE-CFTR) was shown to increase the abundance of CFTR protein [19–21]. Silencing of MARCH2 in CFBE-CFTR cells with a MARCH2 siRNA (Hs_MARCH-II_1) substantially increased the expression of the CFTR protein (Figure 3C and E). Furthermore, CFTR function as measured by forskolin-stimulated short-circuit currents were concurrently increased in MARCH2 silenced cells (Figure 3D and E). Another MARCH2 siRNA (Hs_MARCH-II_3) had similar effect on CFTR protein expression and function (data not shown). Silencing of MARCH2 expression was confirmed by real time RT-PCR measurement of MARCH2 mRNA (Hs_MARCH-II_1 transfected cell is 48.5+/-2.7% of control and Hs_MARCH-II_3 transfected cell is 34.7+/-2.1% of control). Since MARCH2 had a short half-life about 2.5 hours as measured by cycloheximide chase assay of HA-MARCH2 (Figure S2), silencing at mRNA level should closely reflect protein expression. This is especially important as the depletion of MARCH2 protein could not be directly assessed due to poor reactivity of available antibodies. Interestingly, short-lived proteins are frequently found to be regulatory proteins. Taken together, these data suggested that E3 ubiquitin ligase MARCH2 play a role in the regulation of mature CFTR degradation.

### Ubiquitination of CFTR by MARCH2

**Figure 4:**
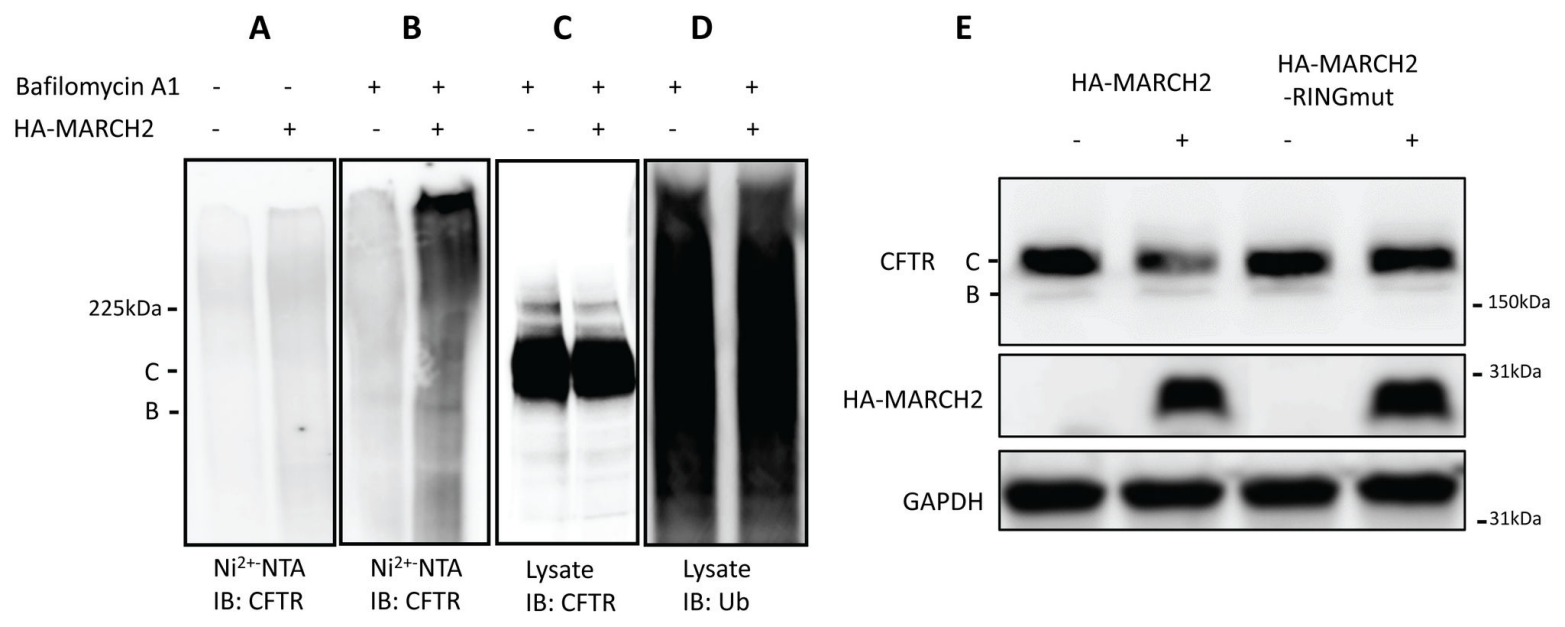
In vivo ubiquitination of CFTR by MARCH2. (**A**) HEK293 cells were first co-transfected with 3µg CFTR (pCMV-CFTR) and 3µg His_6_-ubiquitin. After 24 h, the cells were transfected again with 1µg HA-MARCH2 or the vector plasmid pRK5KS. Twenty-four hours after the second transfection, cell lysates were harvested under denaturing conditions (in a buffer containing 6 M guanidinium hydrochloride). Ubiquitinated proteins were affinity-purified with Ni^2+-^NTA-agarose beads, eluted with Laemmli buffer, separated on SDS-PAGE, transferred to a PVDF membrane, and subjected to the immunoblot analysis with anti-CFTR mouse monoclonal antibody. (**B**) Same as in (A) except cells were treated with 400nM bafilomycin to inhibit lysosome activity 24 hours before harvest under denaturing conditions. Ubiquitinated proteins were affinity-purified with Ni^2+-^NTA-agarose beads and subjected to the immunoblot analysis with anti-CFTR mouse monoclonal antibody. (**C**) Same as in (B) total cell lysates were subjected to immunoblot analysis with anti-CFTR antibody (**D**) The PVDF membrane in (C) was stripped and blotted with an anti-ubiquitinated proteins antibody FK2. Data shown are representative of at least three independent experiments. (**E**) HEK293 cells were co-transfected with 3µg CFTR (pCMV-CFTR) and 1µg HA-MARCH2 or HA-MARCH2 RING. Forty-eight hours after transfection, cell lysates were harvested and subjected to Western blot analysis with anti-CFTR mouse monoclonal antibody 217. Data shown are representative of at least three independent experiments.

To determine whether MARCH2 could ubiquitinate CFTR, we conducted in vivo ubiquitination assays in HEK293 cells. The cells were first transfected with the CFTR and 6xHis-ubiquitin constructs, and 24 h later, they were transfected with an HA-MARCH2 construct. Twenty-four hours after cells were transfected with HA-MARCH2, cell lysates were harvested under denaturing conditions. Ubiquitinated proteins were affinity-purified with Ni^2+-^NTA-agarose beads, eluted with Laemmli buffer, separated on an SDS-PAGE gel, and detected with an anti-CFTR mouse monoclonal antibody. CFTR was minimally ubiquitinated in the absence or in the presence of HA-MARCH2 (Figure 4A). Previously, we have shown that bafilomycin A1 inhibits CAL-mediated lysosomal degradation of CFTR [19,21]. When bafilomycin A1 was added in the culture medium, ubiquitinated CFTR was detected in the presence of HA-MARCH2 (Figure 4B). In addition, bafilomycin A1 was effective in blocking the MARCH2-induced degradation of CFTR (Figure 4C). Overexpression of HA-MARCH2 had no effect on the total cellular ubiquitinated proteins (97.8+/-1.9% of control, p>0.05) (Figure 4D) or Ni^2+^-NTA-agarose bound ubiquitinated proteins (101.0+/-9.4% of control, p>0.05). To further assess the role of ubiquitination in CFTR degradation, we used a MARCH2 RING mutant that lacks catalytic activity. For this purpose, we co-transfected HEK293 cells with wild-type HA-MARCH2 or HA-MARCH2-RINGmut construct where the catalytic sites C64C67 were substituted with serine residues. At 48 h post-transfection, the total protein lysates were harvested, and the CFTR protein level was measured by immunoblot analysis. As shown in Figure 4E, overexpression of HA-MARCH2, but not the HA-MARCH2-RINGmut, reduced the level of CFTR protein.

### The role of the PDZ domain in the MARCH2-mediated degradation of CFTR

**Figure 5:**
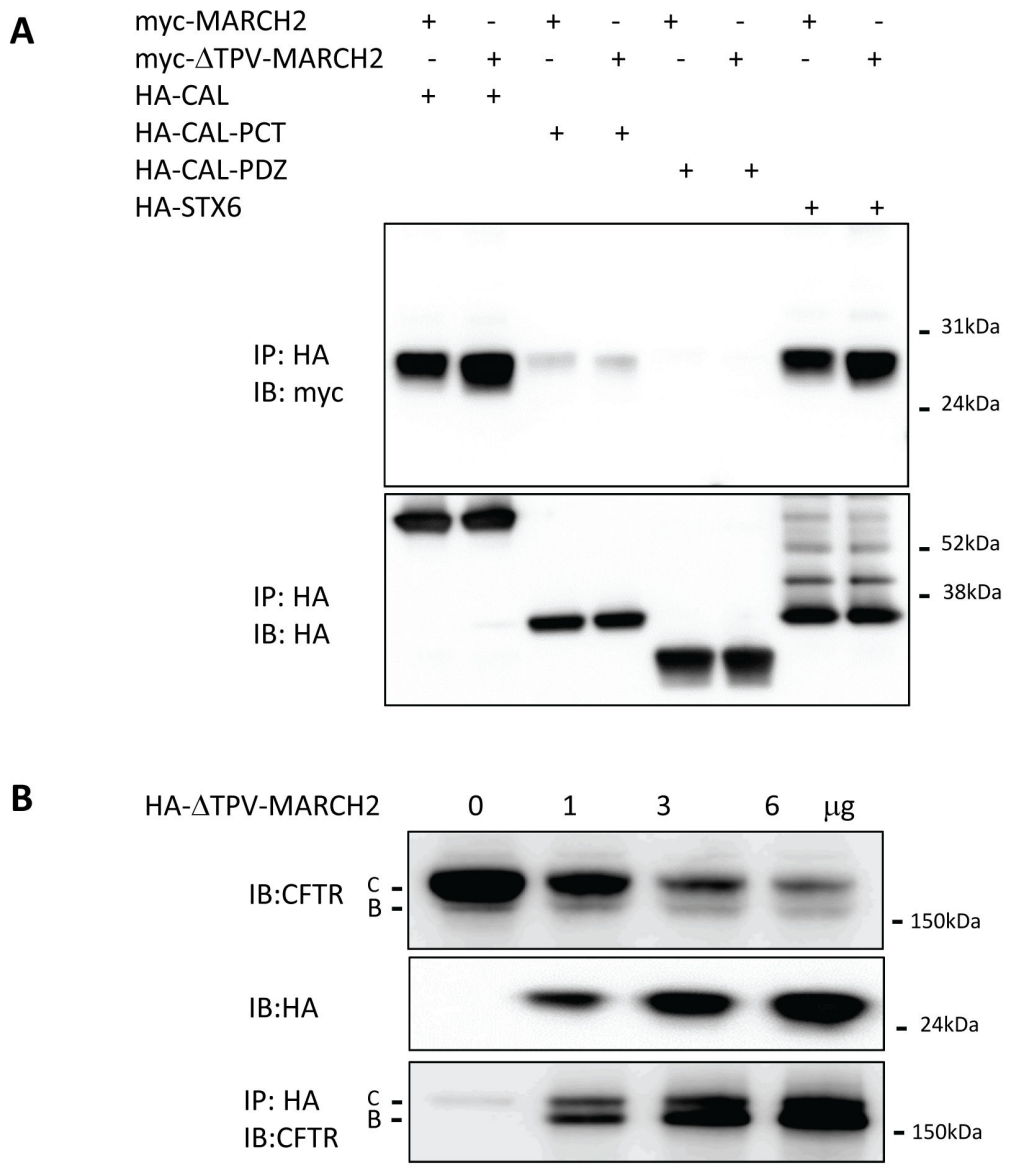
The PDZ binding motif of MARCH2 is not required for MARCH2 degradation of CFTR. (**A**) HEK293 cells were co-transfected with 3µg myc-MARCH2 or myc-ΔTPV-MARCH2 together with 3µg of HA-STX6 or several HA-CAL construct (see below), as indicated. After 48h, cell lysates were harvested and immunoprecipitated with an HA-affinity matrix. Cell lysates and immunoprecipitated materials were subjected to immunoblot analysis with the indicated antibodies. HA-CAL constructs used: HA-CAL (HA-tagged full length CAL), HA-CAL-PDZ (the HA-tagged PDZ domain of CAL) and HA-CAL-PCT (the HA-tagged PDZ and carboxyl domains of CAL) [18] (**B**) HEK293 cells were co-transfected with 3µg CFTR (pCMV-CFTR) and increasing amounts of HA-ΔTPV-MARCH2 as indicated. After 48 h, cell lysates were harvested and subjected to immunoblot analysis with the indicated antibodies. Data shown are representative of at least three independent experiments.

The C-terminal type I PDZ binding motif of CFTR binds to PDZ domain-containing proteins such as NHERF1 and CAL [18,36–38]. The C-terminus of MARCH2 also conforms to a type I PDZ binding motif. It was therefore important for us to determine whether its PDZ binding motif is involved in binding to CAL and in degrading CFTR. To this end, we co-transfected HEK293 cells with the myc-tagged full-length MARCH2 (myc-MARCH2) or a mutant MARCH2 lacking the PDZ binding motif (myc-ΔTPV-MARCH2) construct together with HA-tagged full-length CAL (HA-CAL) or a truncated CAL containing the PDZ-domain and the C-terminus (HA-CAL-PCT) or just the PDZ-domain (HA-CAL-PDZ) [18] and then performed co-immunoprecipitation assays. We found that both myc-MARCH2 and myc-ΔTPV-MARCH2 interacts strongly with full length HA-CAL, weakly with HA-CAL-PCT but not with HA-CAL-PDZ (Figure 5A). Consistent with these co-immunoprecipitation data, we found that HA-ΔTPV-MARCH2 was effective in degrading CFTR (Figure 5B). In addition, HA-ΔTPV-MARCH2 was found to associate with both C and B bands of CFTR which is consistent with the previous finding that MARCH2 PDZ motif mutant is localized in both Golgi and ER [24,25].

**Figure 6:**
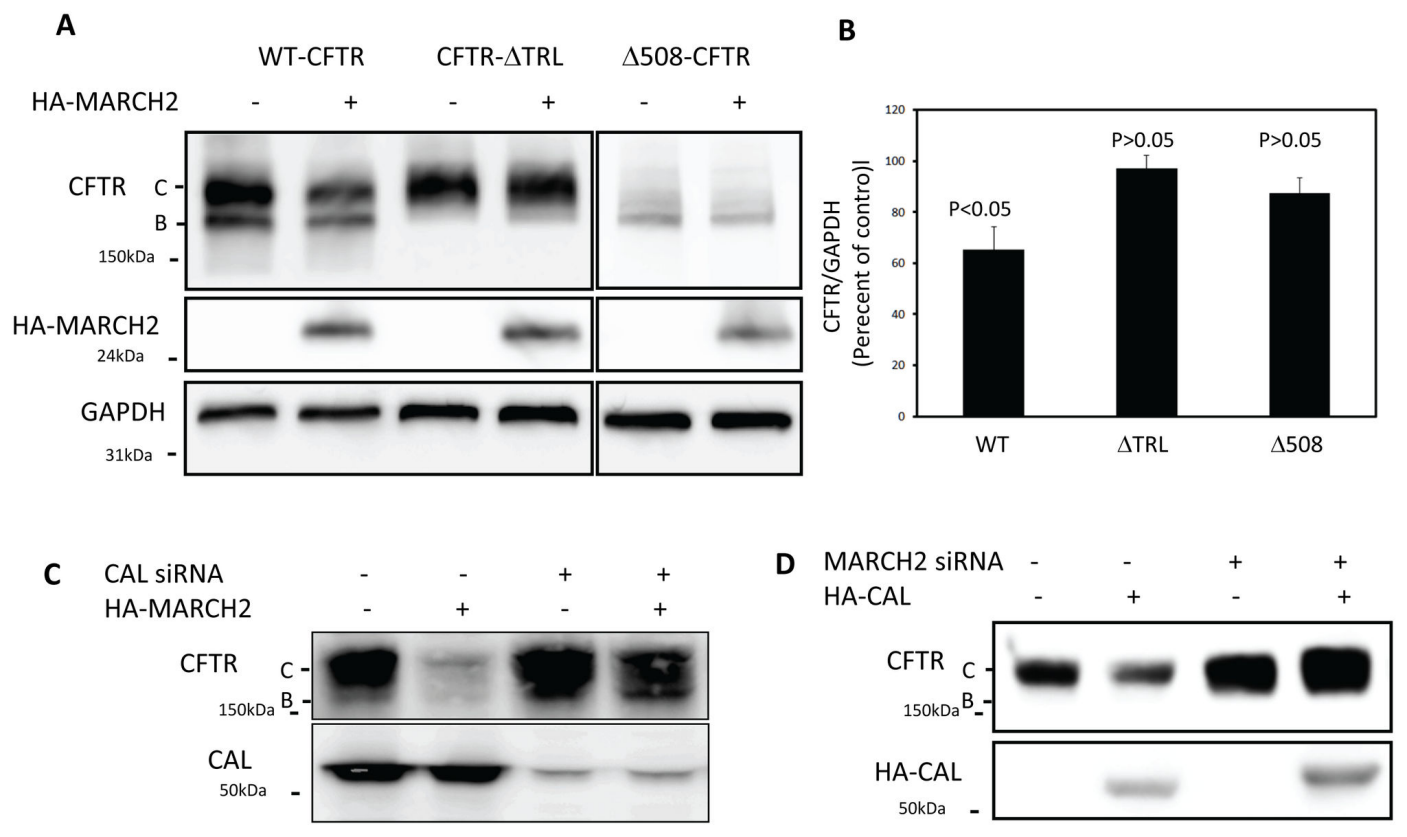
Degradation of wild-type CFTR, but not *Δ*TRL or Δ508 CFTR, by MARCH2. (**A**) HEK293 cells were co-transfected with 3 µg GFP-tagged wild-type (WT), ΔTRL, or Δ508 CFTR and 1µg HA-MARCH2 as indicated. After 48 h, cell lysates were harvested and subjected to immunoblot analysis. (**B**) Densitometric analysis of CFTR in (A). CFTR is normalized to GAPDH. Values are presented as mean +/- S.E. (**C**) HEK293 cells were first transfected with 20 nM CAL siRNA. After 24 h, the cells were transfected with 3µg pCMV-CFTR, with or without 1µg HA-MARCH2, as indicated. At 48 h after the second transfection, cell lysates were collected and subjected to SDS-PAGE and immunoblot analysis with the indicated antibodies. (**D**) HEK293 cells were first transfected with 20 nM MARCH2 siRNA. After 24 h, the cells were transfected with 3µg pCMV-CFTR, with or without 3µg HA-CAL, as indicated. At 48 h after the second transfection, cell lysates were collected and subjected to SDS-PAGE and immunoblot analysis with the indicated antibodies. Data shown are representative of at least three independent experiments.

We then test the role of PDZ-mediated interaction of CFTR and CAL in MARCH2 degradation of CFTR. To this end, we co-transfected HEK293 cells with the HA-MARCH2 construct and either the PDZ-deleted (GFP-ΔTRL-CFTR) or full-length CFTR (GFP-WT-CFTR) construct and then assayed CFTR protein. At 48 h after transfection, the levels of GFP-WT-CFTR, but not GFP-ΔTRL-CFTR, were reduced by HA-MARCH2 (Figure 6A and B). Since GFP-ΔTRL-CFTR does not interact with CAL [18], we then asked whether the presence of CAL was required for degradation. As shown in Figure 6C, silencing of CAL dramatically inhibited the effect of HA-MARCH2 in degrading CFTR, confirming that CAL is indeed required. In order to verify the post-ER site of action that was suggested by MARCH2’s Golgi localization, we took advantage of the CFTR mutant ΔF508-CFTR, which is localized to the ER. As expected, overexpression of HA-MARCH2 had no effect on the level of ΔF508-CFTR abundance (Figure 6A and B). Finally, we tested whether MARCH2 is required for CAL-mediated degradation of CFTR. As shown in Figure 6D, silencing of MARCH2 inhibited the degradation of CFTR by CAL.

### Localization and interaction of MARCH2 with CFTR

**Figure 7:**
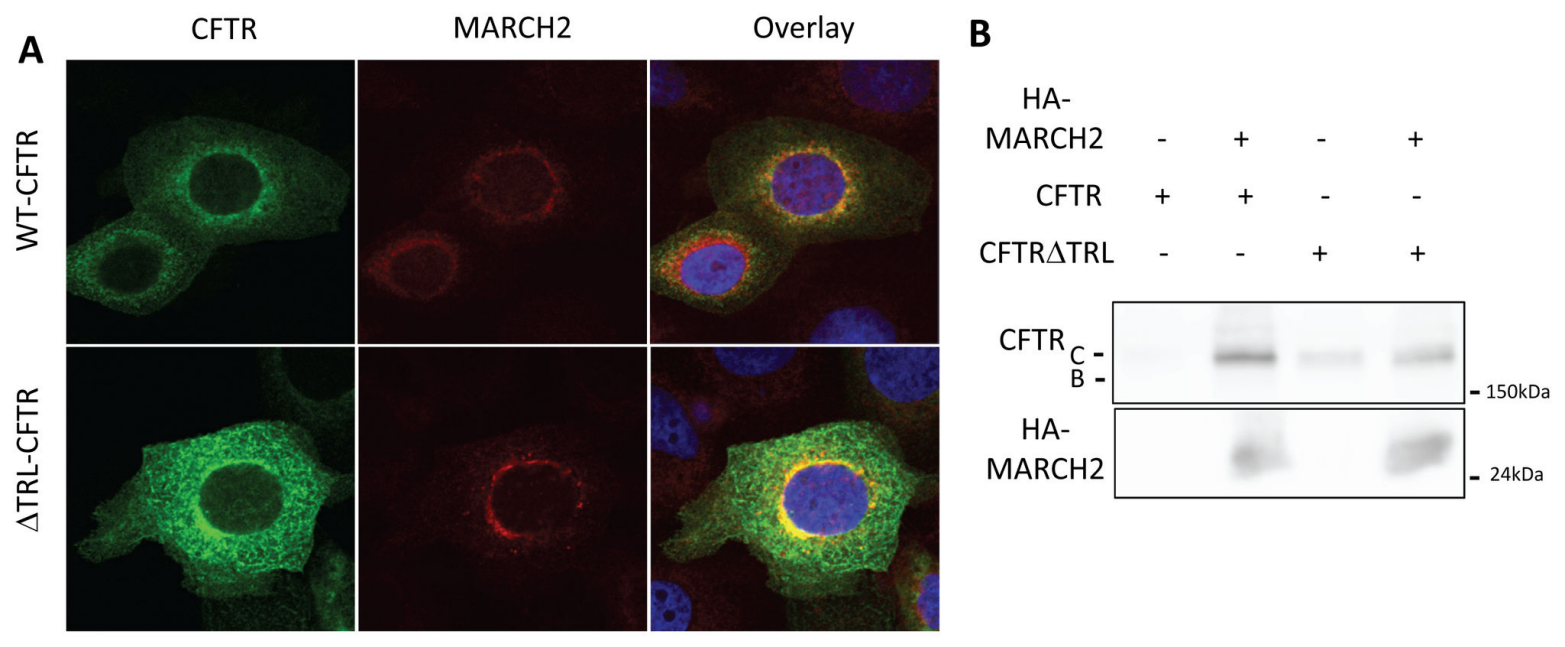
Localization and interaction of MARCH2 with wild-type and *Δ*TRL CFTR. (**A**) CBFE cells grown on coverslips were co-transfected with 3µg GFP-CFTR or 3µg GFP-ΔTRL-CFTR and 0.5 µg HA-MARCH2. After 24h, the cells were fixed and subjected to indirect fluorescent immunocytochemical staining with an anti-HA mouse monoclonal antibody and anti-GFP rabbit polyclonal antibody. HA-MARCH2 appears red and GFP-CFTR green. (**B**) HEK293 cells were co-transfected with 3 µg GFP-CFTR or 3µg GFP-ΔTRL-CFTR and 1 µg HA-MARCH2, as indicated. After 48 h, cell lysates were harvested and immunoprecipitated with an anti-HA affinity matrix. Cell lysates and immunoprecipitated materials were subjected to immunoblot analysis with the indicated antibodies. Data shown are representative of at least three independent experiments.

We then examined the localization of MARCH2 and CFTR in CFBE cells by co-trasnsfecting cells with the HA-MARCH2 and GFP-CFTR constructs as well as a CFTR mutant that lacks the PDZ binding motif (GFP-CFTRΔTRL). At 24h post-transfection, the cells were fixed, permeabilized, subjected to indirect fluorescent immunostaining, and visualized with a laser confocal microscope. As shown in Figure 7A, co-localizations with HA-MARCH2 were observed for both GFP-CFTR and GFP-CFTRΔTRL. Similar results were observed in HEK293 cells co-transfected with HA-MARCH2 and GFP-CFTR or GFP-CFTRΔTRL (Figure S3). We also examined the interactions of MARCH2 and CFTR by performing co-immunoprecipitation assays in HEK293 cells transfected with the HA-MARCH2 and GFP-CFTR or GFP-CFTRΔTRL constructs. As shown in Figure 7B, wild type CFTR (GFP-CFTR) has more robust interaction with HA-MARCH2. Thus localization of CFTR in the Golgi compartment which is independent of PDZ binding motif is necessary but not sufficient for degradation. MARCH2-mediated degradation of CFTR requires not only co-localization but also robust interaction with MARCH2. 

## Discussion

**Figure 8:**
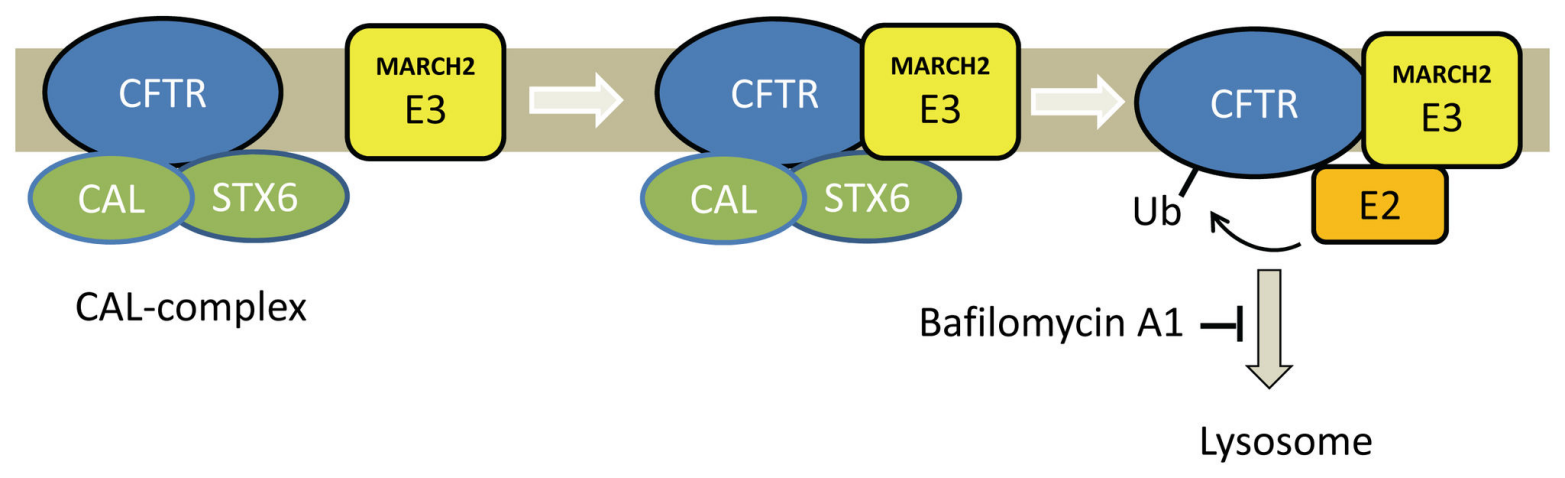
Model of ubiquitination of CFTR by the E3 ligase MARCH2. The CAL complex is formed by the PDZ domain-mediated interaction of CAL and CFTR and the coiled-coil domain-mediated interaction between STX6 and CAL. The CAL complex recruits the E3 ubiquitin ligase MARCH2 through an interaction between STX6 and MARCH2. The recruitment of MARCH2 leads to the association, ubiquitination and degradation of CFTR in the lysosome.

In this paper, we have identified a Golgi-localized E3 ubiquitin ligase, MARCH2, that associates with CAL complexes and mediates the lysosomal degradation of CFTR. Previously, the Golgi-localized CAL complex had been shown to associate with CFTR and promote its degradation in the lysosomes, but the molecular mechanism by which the binding of CAL and STX6 led to CFTR degradation was unknown. Several lines of evidence presented here support the contention that MARCH2 plays a role in the CAL complex-mediated degradation of CFTR: (1) MARCH2 co-localized with and interacts with the CAL complex. (2) MARCH2 associated with CFTR as well as with CAL and STX6. This association of MARCH2 with CFTR was dependent on the presence PDZ motif of CFTR(3). MARCH2 ubiquitinated CFTR in an *in vivo* ubiquitination assay(4). Overexpression of wild-type MARCH2, but not the catalytically dead RING mutant of MARCH2, dramatically reduced the steady-state protein levels of CFTR, which could be blocked by bafilomycin A1 treatment(5). Silencing of CAL ablated MARCH2’s effect on CFTR protein expression, and MARCH2 had no effect on a CFTR mutant missing the PDZ binding motif(6). Silencing of endogenous MARCH2 increased the level of CFTR protein expression in a CF bronchial epithelial cell line. These data suggest a model in which the recruitment of MARCH2 E3 ligase to the CAL complex leads to the association, ubiquitination and degradation of CFTR (Figure 8).

Members of the family of membrane-associated RING-CH domain ubiquitin E3 ligases have two to four membrane-spanning domains, consistent with their role in the ubiquitination of the membrane protein CFTR. Interestingly, other E3 ligases that ubiquitinate CFTR (HRD1, RMA1, CHIP, and gp78), with the exception of CHIP, are also membrane-bound. MARCH family proteins are the cellular homologs of the viral immune evasion proteins of the K3 family. When a panel of known substrates of viral K3 family proteins was tested, almost all MARCH members were found to degrade some cell-surface glycoproteins. For instance, MARCH2 was found to degrade TfR and B7.2, but not MHC I [30]. Whether they represent bona fide targets of MARCH2 still requires further investigation, especially by siRNA-mediated silencing to rule out potential nonspecific effects of the overexpression system. In addition, it is unknown whether MARCH2 binds to TfR and B7.2, or whether MARCH2 ubiquitinates these proteins. In another report, overexpression of MARCH2 was found to lead to a reduction in cell-surface transferrin receptor without affecting total cellular transferrin receptor expression. MARCH2 overexpression does not affect the degradative transport pathway to lysosomes in general, and the lysosomal degradation of EGFR was unaffected by MARCH2 overexpression. In contrast, MARCH2 overexpression reduced the overall abundance of CFTR by promoting its lysosomal degradation. These data argue for a specificity of interaction of MARCH2 with CFTR through its association with the CAL complex, which acts as an adaptor to recruit MARCH2 to its substrate, CFTR. In addition to MARCH2, MARCH3 also interacts with and co-localizes with STX6 at the level of the Golgi. This observation is not surprising, since MARCH2 and MARCH3 are the most closely related proteins in the MARCH family, with 63% identity in protein sequence [25]. MARCH2 is ubiquitously expressed in all human tissues examined, whereas MARCH3 is enriched in the lungs, colon, and spleen [24]. We found that overexpression of MARCH3 led to a dose-dependent degradation of CFTR (unpublished observation), and we suggest that MARCH3 may play similar role in regulating CFTR degradation in the lungs, colon, and spleen.

Our data suggest that the spatial co-localization and physical interaction of MARCH2 with CFTR complexes determine the specificity of its function. The subcellular localization of MARCH2 in the Golgi and endosomes suggests that its contribution to the degradation of CFTR occurs in the post-ER compartment. Consistent with this conclusion, MARCH2 had no effect on the degradation of ER-localized ΔF508-CFTR (Figure 6). However, co-localization in the same compartment is necessary but not sufficient for the degradation as MARCH2 had no effect on the degradation of ΔTRL-CFTR (Figure 6 and Figure 7). Robust interaction of WT CFTR with MARCH2 is also required to its degradation. The Golgi-localized, CFTR-associated protein CAL retains and degrades mature CFTR in lysosomes [18,19]. CAL binds to CFTR through a PDZ domain-mediated interaction. Disruption of this interaction between CAL and CFTR by either silencing CAL or deletion of PDZ binding motif (ΔTRL) inhibited MARCH2-mediated degradation of CFTR (Figure 6). Disruption of this interaction between CAL and CFTR with synthetic peptide inhibitors prolongs the half-life of mature CFTR and restores that of ΔF508 CFTR [22]. The coiled-coil domains of CAL are critical in recruiting accessory proteins that link CFTR-CAL to the cellular trafficking machinery. The binding to STX6 and MARCH2 leads to the ubiquitination and degradation of CFTR. Therefore, CAL acts as adaptors for the E3 ligase MARCH2 in ubiquitinating its substrate, CFTR. Like many E3 ligases (e.g., CHIP), MARCH2 may have multiple cellular targets and unique functions, and its interaction with the CAL complex differentiates its role in the degradation of CFTR from that of other targets. CAL regulates the post-Golgi trafficking of CFTR, either toward the lysosome or toward the plasma membrane, depending on the components of the complexes. CAL also binds to the Rho small GTPase TC10 via its coiled-coil domains [39,40]. Binding to the GTP-loaded TC10 promotes CFTR trafficking to the cell surface [41]. It would be interesting to investigate whether the binding to the GTP-loaded TC10 affects the binding and/or activity of MARCH2 toward CFTR.

The most prevalent CF mutation, the ΔF508 mutation, is present in >70% of CF patients who lack functional mature CFTR because their mutant CFTR is eliminated by cellular quality control systems [42–44]. In the case of ΔF508 and other protein-folding mutants, therapeutic strategies that generate mature CFTR partially restore CFTR function. Understanding all aspects of the regulation of mature CFTR turnover is therefore important for developing effective CF treatments. Recently, several novel small molecule drug candidates for treating CF have emerged from high-throughput screenings [45–48]. Ivacaftor (VX-770) is a CFTR potentiator that has been approved in the US for the treatment of CF patients carrying G551D mutations [49]. While VX770 potentiates the function of CFTR molecules that are already at the plasma membrane, VX-809 is an investigational drug that targets the processing of ΔF508 CFTR [47]. In a cell model, VX-809 enhanced chloride secretion to approximately 14% of that in non-CF human bronchial epithelial cells. The phase IIa study of VX-809 in subjects with CF homozygous for the ΔF508 CFTR mutation showed only limited clinical efficacy in the respiratory tract [50]. Additional intervention may be required to restore the abundance and/or function of mature ΔF508 to a clinically meaningful level. It is interesting to note that silencing of MARCH2 has modest effect in stabilizing temperature-rescued ΔF508 CFTR in Hela cells but not in IB3 cells [12]. These results are consistent with our finding that MARCH2 play a role in promoting the degradation of mature but not immature CFTR. Furthermore, the role of endogenous MARCH2 in regulating CFTR protein abundance and function was confirmed in CFBE cells. Thus modulation of the CAL/STX6/MARCH2-mediated degradation of mature CFTR offers another promising area for potential intervention. 

## Supporting Information

Figure S1 Co-localization of MARCH2 with *Golgiprotein* Golgin 160. HEK293 cells grown on coverslips were transfected with 1µg HA-MARCH2. After 24 h, cells were fixed and subjected to indirect fluorescent immunocytochemical staining with an anti-HA mouse monoclonal antibody and an anti-Golgin160 rabbit polyclonal antibody followed by goat anti-mouse Alexa Fluor 488-conjugated secondary antibody and goat anti-rabbit Alexa Fluor 594-conjugated secondary antibody and. HA-MARCH2 appears green and Golgin160 red. Data shown are representative of at least three independent experiments.
(TIF)

Figure S2 Cycloheximide chase assay of HA-MARCH2. (**A**) HEK293 cells were transfected with 1µg HA-MARCH2. After 24 h, 100µg/ml cycloheximide (CHX) was added to the culture medium and cell lysates were harvested at indicated times after the CHX addition. Cell lysates were then subjected to immunoblot analysis. (**B**) Densitometric analysis of CFTR normalized to GAPDH expression in (A). Values are presented as mean +/- S.E.
(TIF)

Figure S3 Localization of MARCH2 with wild-type and *Δ*TRL CFTR. HEK293 cells grown on coverslips were co-transfected with 3µg GFP-CFTR or 3µg GFP-ΔTRL-CFTR and 0.5 µg HA-MARCH2. After 24 h, the cells were fixed and subjected to indirect fluorescent immunocytochemical staining with an anti-HA mouse monoclonal antibody and an anti-GFP rabbit polyclonal antibody followed by goat anti-rabbit Alexa Fluor 488-conjugated secondary antibody and goat anti-mouse Alexa Fluor 594-conjugated secondary antibody. HA-MARCH2 appears red and GFP-CFTR green. Data shown are representative of at least three independent experiments.
(TIF)
