# Supplementary figures and images for: Ubiquitination and Degradation of CFTR by the E3 Ubiquitin Ligase MARCH2 through Its Association with Adaptor Proteins CAL and STX6
Source: PLoS One. 2013 Jun 20;8(6):e68001. doi: 10.1371/journal.pone.0068001 (PMC3688601; doi:10.1371/journal.pone.0068001)

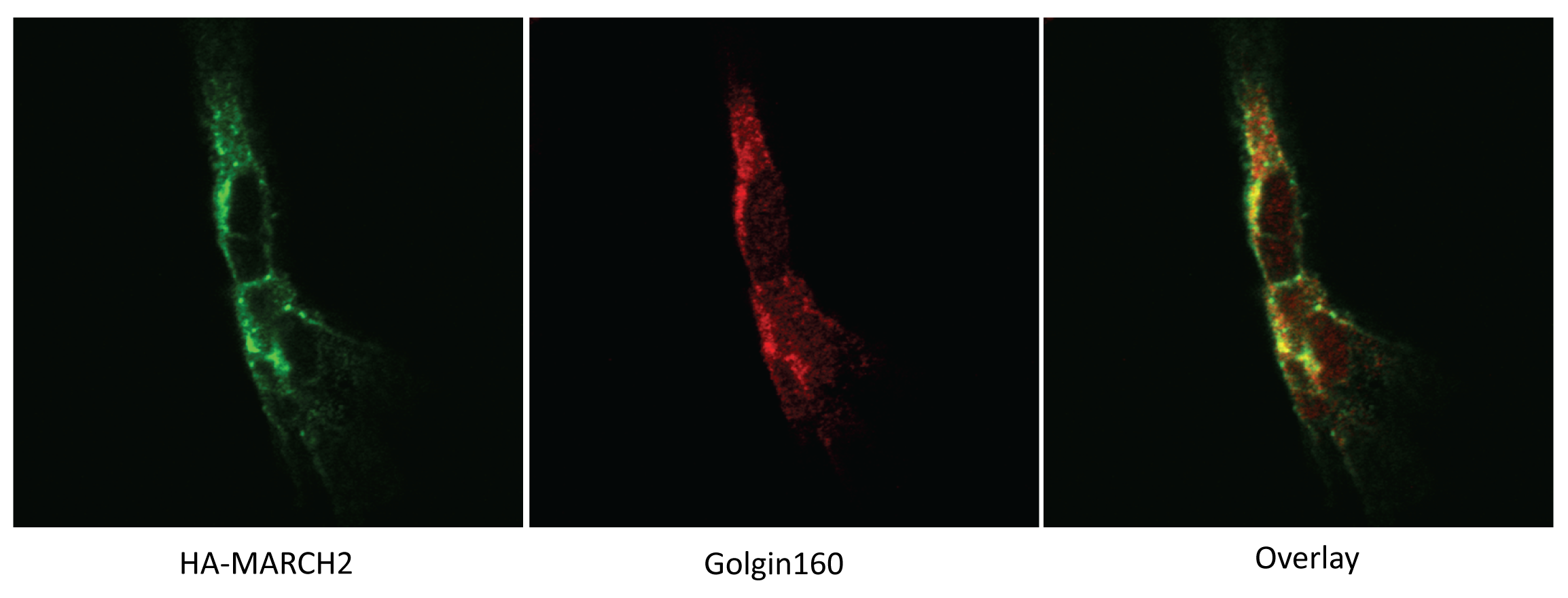

Supplement: Figure S1 — HEK293 cells grown on coverslips were transfected with 1µg HA-MARCH2. After 24 h, cells were fixed and subjected to indirect fluorescent immunocytochemical staining with an anti-HA mouse monoclonal antibody and an anti-Golgin160 rabbit polyclonal antibody followed by goat anti-mouse Alexa Fluor 488-conjugated secondary antibody and goat anti-rabbit Alexa Fluor 594-conjugated secondary antibody and. HA-MARCH2 appears green and Golgin160 red. Data shown are representative of at least three independent experiments. (TIF) [file pone.0068001.s001.tif]

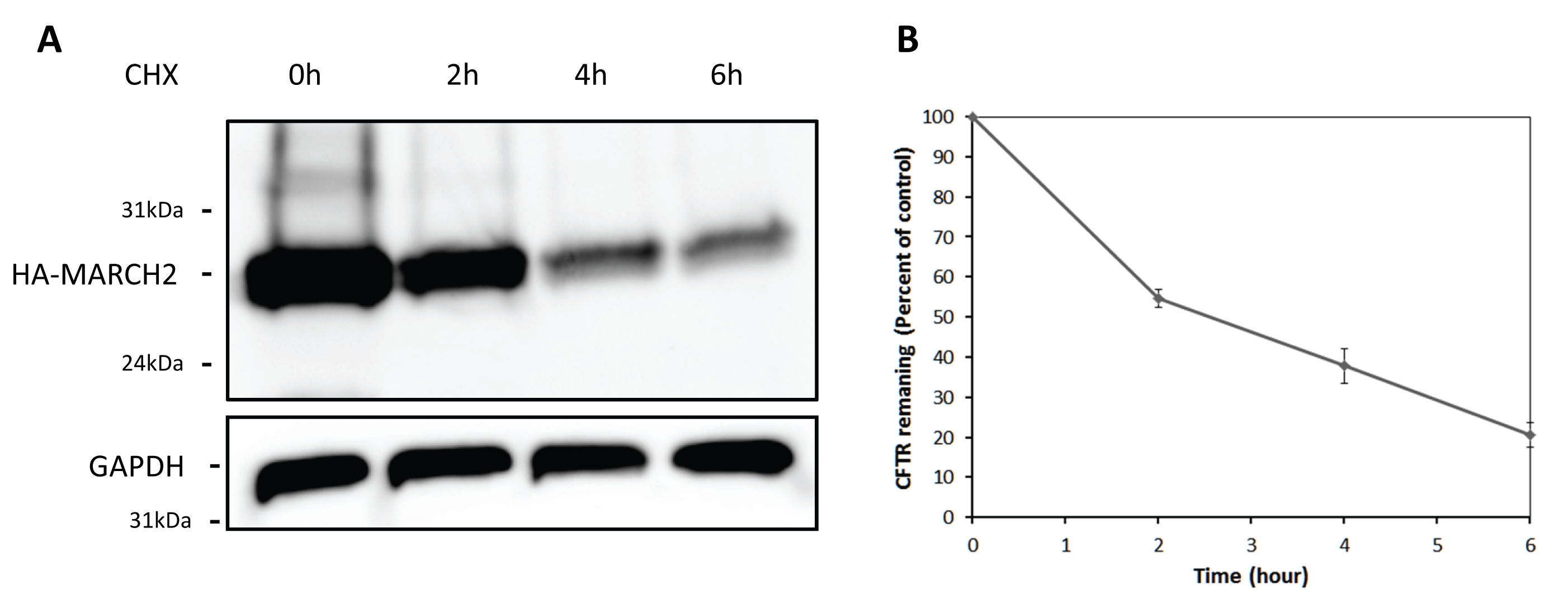

Supplement: Figure S2 — (A) HEK293 cells were transfected with 1µg HA-MARCH2. After 24 h, 100µg/ml cycloheximide (CHX) was added to the culture medium and cell lysates were harvested at indicated times after the CHX addition. Cell lysates were then subjected to immunoblot analysis. (B) Densitometric analysis of CFTR normalized to GAPDH expression in (A). Values are presented as mean +/- S.E. (TIF) [file pone.0068001.s002.tif]

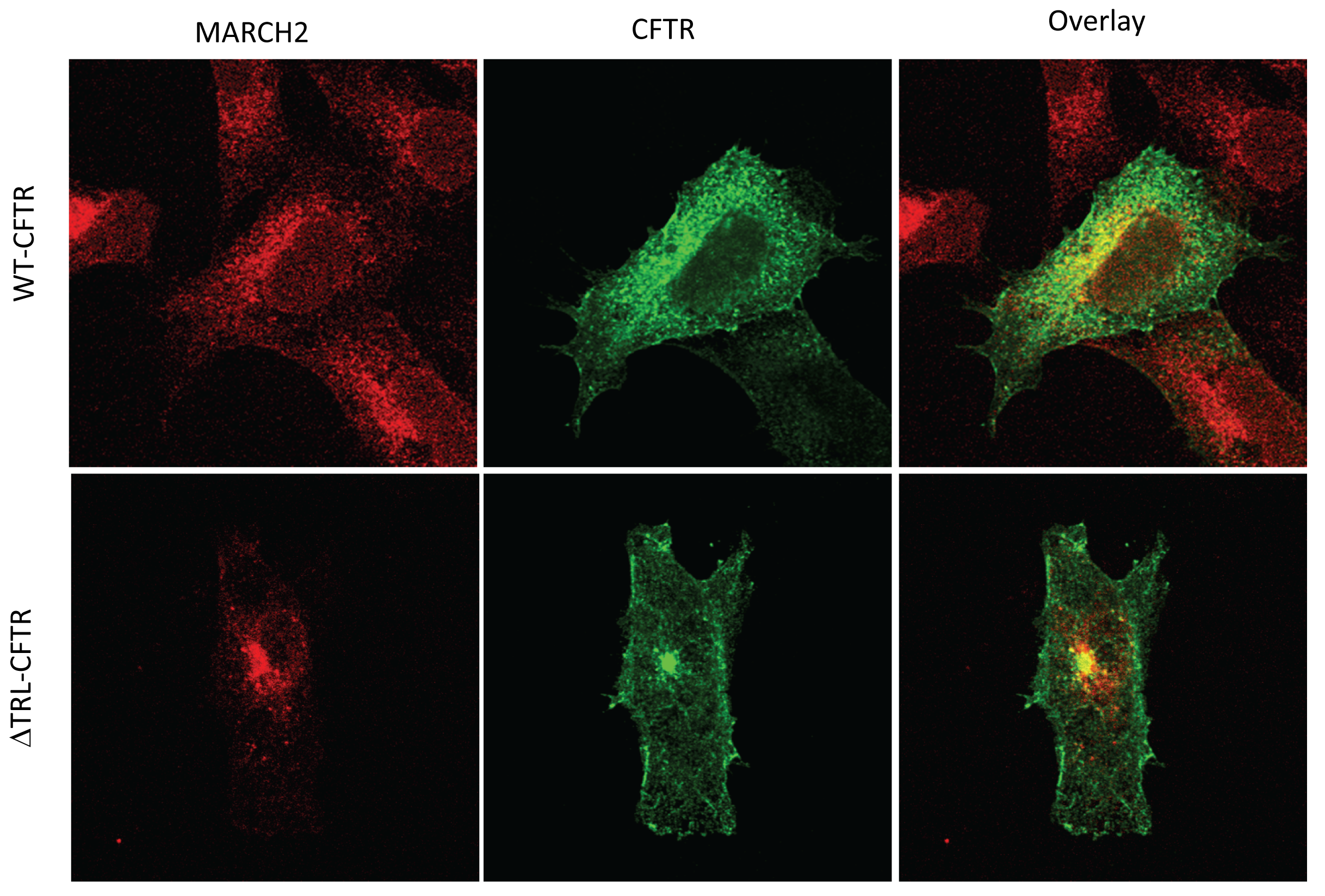

Supplement: Figure S3 — HEK293 cells grown on coverslips were co-transfected with 3µg GFP-CFTR or 3µg GFP-ΔTRL-CFTR and 0.5 µg HA-MARCH2. After 24 h, the cells were fixed and subjected to indirect fluorescent immunocytochemical staining with an anti-HA mouse monoclonal antibody and an anti-GFP rabbit polyclonal antibody followed by goat anti-rabbit Alexa Fluor 488-conjugated secondary antibody and goat anti-mouse Alexa Fluor 594-conjugated secondary antibody. HA-MARCH2 appears red and GFP-CFTR green. Data shown are representative of at least three independent experiments. (TIF) [file pone.0068001.s003.tif]
